# Staged Management of Extensive Suprapubic Wound Necrosis After Hysterectomy Using Negative Pressure Wound Therapy and Delayed Skin Grafting

**DOI:** 10.1111/iwj.71021

**Published:** 2026-08-25

**Authors:** Stefano Restaino, Edoardo Farnè, Martina Arcieri, Marta Bortolozzo, Anna Momic, Tina Moni Bidin, Beatrice Nonino, Irene Silvestri, Glenda Caputo, Marco D'Asta, Marco Petrillo, Guglielmo Stabile, Lorenza Driul, Giuseppe Vizzielli

**Affiliations:** ^1^ Clinic of Obstetrics and Gynecology, “Santa Maria Della Misericordia” University Hospital Azienda Sanitaria Universitaria Friuli Centrale Udine Italy; ^2^ PhD School in Biomedical Sciences, Gender Medicine, Child and Women Health, University of Sassari Sassari Italy; ^3^ Department of Medicine (DMED) University of Udine Udine Italy; ^4^ Plastic and Reconstructive Surgery Department Azienda Sanitaria Universitaria Friuli Centrale Udine Italy; ^5^ Department of Obstetrics and Gynecology ARNAS Garibaldi Hospital Catania Italy; ^6^ Department of Medicine, Surgery and Pharmacy University of Sassari Sassari Italy; ^7^ Department of Medical and Surgical Sciences, Institute of Obstetrics and Gynecology University of Foggia Foggia Italy

**Keywords:** complex wounds, gynaecologic surgery, multidisciplinary approach, negative pressure wound therapy, surgical wound dehiscence

## Abstract

Postoperative wound complications are a major clinical challenge, especially in patients with comorbidities such as overweight and diabetes mellitus. Extensive abdominal wall necrosis after gynaecologic surgery is uncommon but may lead to prolonged healing, increased morbidity and the need for complex reconstructive procedures. Negative pressure wound therapy (NPWT) is an effective option for complex surgical wounds, promoting granulation tissue, improving perfusion and reducing bacterial burden. This report describes the case of a 58‐year‐old woman with overweight status and Type 2 diabetes mellitus who developed extensive suprapubic wound dehiscence with associated tissue necrosis following hysterectomy and bilateral salpingo‐oophorectomy performed for a giant uterine leiomyoma. Following surgical debridement, NPWT was started and maintained with scheduled dressing changes. Progressive improvement was observed, with wound size decreasing from about 20 to 10 cm in 30 days and robust granulation tissue formation. Once the wound bed was optimised, definitive closure was achieved with an autologous skin graft. This case supports the role of NPWT as a bridge therapy in managing complex postoperative gynaecologic wounds in high‐risk patients. A multidisciplinary approach involving gynaecologists, plastic surgeons and wound care specialists was essential for a favourable outcome. NPWT should be considered a valuable option for selected gynaecologic patients with extensive wound complications.

## Highlights

- NPWT demonstrates high efficacy in complex gynaecologic wound necrosis post‐hysterectomy.
- After debridement, therapy rapidly reduces wound size from 20 to 10 cm in 30 days.
- Granulation tissue forms effectively, enabling autologous skin graft closure after 36 days.

## 1 Introduction

Uterine leiomyomas are the most common benign tumours of the female reproductive tract and represent a frequent indication for hysterectomy, particularly, in peri‐ and postmenopausal women [1]. Although minimally invasive approaches are preferred whenever feasible, the surgical management of giant uterine leiomyomas often requires laparotomy due to uterine size, distorted anatomy or technical limitations [2]. Open abdominal surgery, especially when associated with wide transverse or extended suprapubic incisions, is known to increase the risk of postoperative wound complications [3].

Surgical site complications—including wound dehiscence, infection and tissue necrosis—are particularly prevalent in patients with risk factors such as obesity, overweight status, diabetes mellitus and vascular comorbidities [4]. These conditions impair tissue perfusion, inflammatory response and collagen synthesis, ultimately delaying wound healing and increasing the likelihood of complex wound failure [5]. In gynaecologic surgery, extensive abdominal wall necrosis remains a rare but challenging complication, frequently requiring prolonged treatment and multidisciplinary management.

Negative pressure wound therapy (NPWT) has become an established modality for the treatment of complex acute and chronic wounds [6]. By applying controlled subatmospheric pressure, NPWT promotes angiogenesis and granulation tissue formation, reduces edema and bacterial load and facilitates wound contraction [6]. Although widely adopted in general surgery, trauma and obstetrics, its application in gynaecologic postoperative wound complications is less frequently reported [7]. The present case describes the successful use of NPWT as a bridge therapy for extensive suprapubic wound necrosis following hysterectomy for a giant uterine leiomyoma, highlighting its role in optimising wound healing prior to definitive surgical reconstruction.

## 2 Case Presentation

A 58‐year‐old nulliparous woman with a medical history significant for Type 2 diabetes mellitus and overweight status (body mass index 29 kg/m^2^) and chronic lower‐limb varicosities was referred to our gynaecology unit for evaluation of a large uterine mass. The patient initially presented with respiratory symptoms and thoracoabdominal imaging incidentally revealed a voluminous pelvic mass. Subsequent gynaecologic assessment was prompted by an episode of menometrorrhagia.

Physical examination revealed a markedly distended abdomen with a firm, non‐tender mass extending from the pubic region to the xiphoid process. Pelvic ultrasonography demonstrated a large isoechogenic uterine mass with diffuse acoustic shadowing, measuring approximately 35 cm in its largest diameter. Tumour markers were within normal limits, supporting a benign aetiology. After optimisation of glycaemic control and resolution of concomitant infection, surgical treatment was planned.

The patient underwent hysterectomy with bilateral salpingo‐oophorectomy via laparotomy due to the size of the uterus and limited feasibility of a minimally invasive approach. The uterus weighed approximately 3 kg. No intraoperative complications occurred and estimated blood loss was minimal. Histopathological examination confirmed a benign uterine leiomyoma associated with an ovarian cystadenofibroma.

At post‐operative day (POD) 11, wound dehiscence with overlying cutaneous necrosis was identified at the cranial aspect of the prior Pfannenstiel‐type laparotomy incision. The remaining suture line appeared regular and non‐infiltrated; mild superficial haemorrhagic suffusion and an approximately 10‐cm eschar were noted. Laboratory evaluation showed WBC 12.39 × 10^3^/μL and CRP 111.48 mg/L; empiric antibiotics were started and the patient was admitted.

At POD 12, operative exploration confirmed well‐demarcated, malodorous necrosis of the suprapubic abdominal flap (15 × 8 × 3 cm), extending to approximately 2 cm below the umbilicus. Necrosis involved the skin and subcutaneous tissue only; the anterior rectus sheath was intact and adherent, with no fascial dehiscence, exposed fascia, supra‐fascial haematoma, undermining or tunnelling. A single sharp surgical debridement was performed, followed by immediate initiation of NPWT (VAC system). Wound swab cultures were obtained intraoperatively and yielded no growth; no imaging was performed due to lack of clinical suspicion for deeper infection.

NPWT was delivered with intermittent −80 mmHg using black polyurethane foam, with dressing changes approximately every 72 h over an overall course of ~36 days. At each change, the periwound skin was cleansed with normal saline, thoroughly dried and a barrier spray was applied. Weekly outpatient assessments documented progressive wound contraction; at one visit, fibrinous material and erythematous margins were managed with local cleansing and conservative sharp removal of fibrin/necrotic debris, with ongoing improvement.

A multidisciplinary evaluation involving gynaecologists and plastic surgeons led to the decision to proceed to definitive coverage at POD 48 with an autologous split‐thickness skin graft (STSG; 0.45 mm) harvested from the gluteal region. The patient was discharged 4 days post‐grafting on amoxicillin–clavulanic acid 1 g PO three times daily for 7 days, with weekly follow‐up. Graft take was not formally quantified; however, no complications occurred and at 10 weeks the wound was fully epithelialised, closed and stable, with a pink–reddish appearance consistent with neovascularization. No further investigations were performed given complete healing.

## 3 Discussion

**FIGURE 1:**
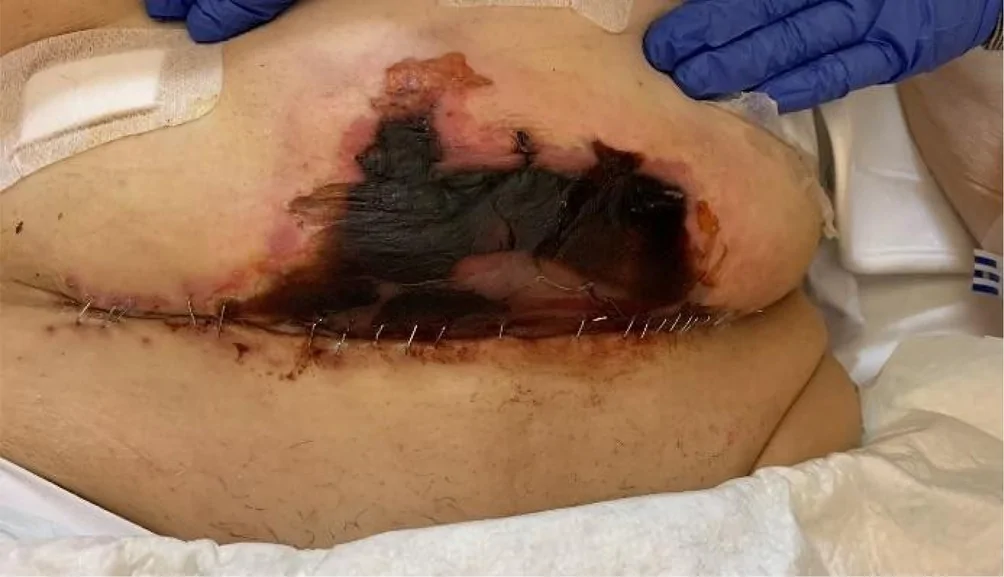
Extensive suprapubic wound dehiscence with skin and subcutaneous tissue necrosis following gynaecologic surgery, after surgical debridement and prior to initiation of negative pressure wound therapy.

**FIGURE 2:**
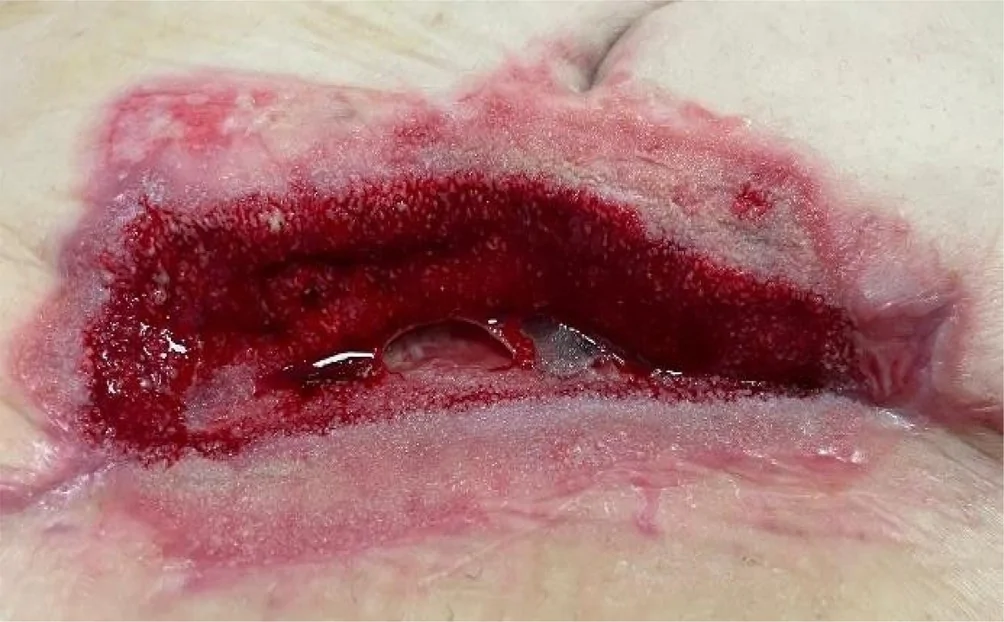
Progressive wound healing approximately 30 days after initiation of negative pressure wound therapy, showing marked reduction in wound size and development of healthy granulation tissue.

**FIGURE 3:**
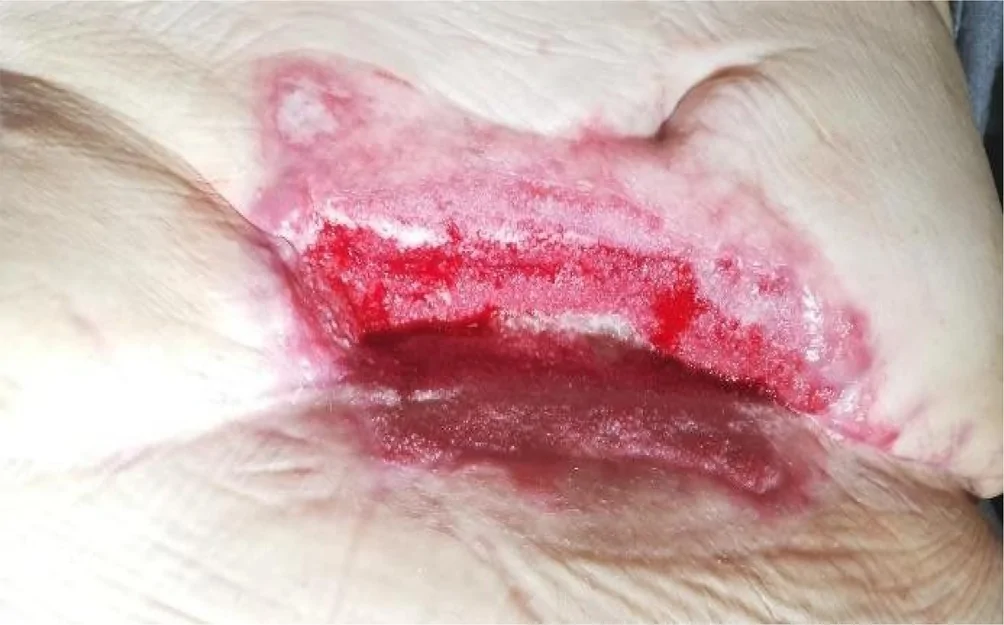
Wound appearance approximately 36 days after initiation of negative pressure wound therapy, with a well‐granulated wound bed suitable for definitive closure with autologous skin grafting.

This case contributes to the limited literature on NPWT in gynaecologic surgery by highlighting several practical aspects of management. First, it illustrates the successful treatment of an unusually extensive suprapubic wound necrosis occurring after hysterectomy in a patient with multiple factors associated with impaired wound healing. Second, it demonstrates the role of NPWT as a bridge therapy, allowing progressive wound contraction and optimisation of the wound bed before definitive reconstruction with autologous skin grafting. In our patient, wound dimensions decreased by approximately 50% over 30 days of treatment, providing an objective measure of response (Figures 1, 2, 3).

From a practical perspective, this case emphasises the importance of early recognition of wound deterioration, prompt surgical debridement, timely initiation of NPWT, regular reassessment during dressing changes and multidisciplinary collaboration involving gynaecologists, plastic surgeons and wound care specialists. Finally, it illustrates how the decision to proceed with skin grafting should be guided not by a predefined timeline but by the achievement of a well‐vascularised, adequately granulated wound bed suitable for definitive closure (Table 1).

**TABLE 1:** Timeline of post‐operative course.

Time point	Findings	Intervention(s)	Outcome
POD 0^a^	Hysterectomy with bilateral salpingo‐oophorectomy via laparotomy	—	—
POD 11	Wound dehiscence; cranial skin necrosis/eschars (~10 cm); WBC 12.39 × 10^3^/μL; CRP 111.48 mg/L	Admission; empiric antibiotics	Planned surgical revision
POD 12	Cutaneous/subcutaneous necrosis 15 × 8 cm extending cranially to ~2 cm below the umbilicus; fascia intact; no hematoma/undermining/tunnelling	Surgical debridement; NPWT started (intermittent −80 mmHg); intraoperative swabs negative	NPWT continued
POD 12–48	Progressive granulation and wound contraction	NPWT; dressing changes ~q72 h; weekly outpatient reviews; conservative debridement as needed	Wound prepared for definitive coverage
POD 48	Wound suitable for closure	STSG 0.45 mm (gluteal donor site)	Definitive coverage performed
POD 50	Stable postoperative course	Discharge; amoxicillin/clavulanate 1 g PO TID × 7 days; weekly follow‐up arranged	Outpatient management
Post‐op Week 10	Follow‐up visit	—	Complete epithelialization; no further complications reported

Abbreviations: CRP, C‐reactive protein; NPWT, negative‐pressure wound therapy; PO, by mouth (per os); POD, post‐operative day; STSG, split‐thickness skin graft; TID, three times daily (ter in die); WBC, white blood cell count.

^a^ POD indicates post‐operative day counted from the day of index surgery (POD 0).

To our knowledge, reports describing NPWT as bridge therapy for extensive suprapubic flap necrosis following hysterectomy for benign gynaecologic disease remain scarce.

NPWT is a well‐established method for managing complex surgical wounds, enhancing granulation tissue, controlling infection and reducing exudate. Its role in gynaecology, although less frequently described, is gaining recognition. Since its introduction in Europe in 1994 and the United States in 1995, NPWT has been employed in the management of acute, chronic, traumatic and post‐operative wounds, as well as ulcers, grafts and burns [8].

According to NICE guidance, prophylactic negative‐pressure wound therapy (NPWT) may be considered to reduce surgical‐site infections in selected high‐risk women undergoing elective caesarean delivery, particularly, those with a BMI ≥ 35 [9]. However, these recommendations are derived from the obstetric setting and may not be directly applicable to gynaecologic surgery.

At present, there are no gynaecology‐specific evidence‐based guidelines addressing the use of NPWT for the management of complex postoperative wound complications [9]. Consequently, its use is generally guided by clinician judgement, multidisciplinary assessment and extrapolation from evidence generated in other surgical specialties. In this context, our case contributes to the limited available literature by supporting NPWT as a potential therapeutic option in carefully selected gynaecologic patients with extensive postoperative wound complications.

Traditional wound management often relies on debridement and secondary intention healing, but these approaches may be insufficient in comorbid patients. NPWT offers distinct advantages by improving local perfusion and reducing bacterial load, thereby accelerating recovery and minimising the risk of chronic complications [10].

Importantly, NPWT served as a bridge therapy, allowing wound optimization before definitive closure with a skin graft [8].

Despite its benefits, NPWT has several contraindications, necessitating a comprehensive wound assessment prior to initiation. Contraindications include exposed vasculature or organs, enteric or non‐enteric fistulae (due to the risk of haemorrhage or fluid imbalance), untreated necrotic tissue or eschar, underlying malignancy and osteomyelitis [11].

When appropriately indicated, NPWT can significantly improve outcomes in complex wounds—shortening hospital stays, facilitating easier wound care and yielding superior healing compared to conventional dressings—despite the higher per‐unit cost [12]. However, its routine use in uncomplicated or low‐risk surgical wounds is not cost‐effective.

According to current literature, in the obstetrics field, NPWT has been extensively applied to manage wound complications in obese patients undergoing caesarean delivery. Literature also documents its use during pregnancy for abdominal wound dehiscence, post‐appendectomy infections and cases such as necrotising fasciitis of the breast. In the postpartum period, NPWT has been employed to treat episiotomy dehiscence and abdominal compartment syndrome secondary to amniotic fluid embolism [13].

In gynaecology, NPWT (or VAC Therapy) has demonstrated favourable outcomes in the management of oncologic surgical wounds, particularly, following total or partial vulvectomy, outperforming traditional dressings [14].

In the present case, it is plausible that the development of necrosis in the suprapubic abdominal flap was likely multifactorial favoured by the extended Pfannenstiel incision due to the large uterine size. In particular, the surgical incision may have compromised the vascular supply by transecting the superficial epigastric artery, whose contribution to the perfusion of the abdominal wall is clinically significant. Loss of this vascular support, in association with the patient's comorbidities (diabetes and overweight status), could have played a key role in the onset and progression of necrosis. This hypothesis is consistent with the anatomical distribution of the lesion and with literature describing the risk of injury to both superficial and deep inferior epigastric vessels during extensive abdominal wall incisions or trocar placement [4].

Reduced local perfusion was a key contextual factor in this case, given the extent and distribution of flap necrosis and the patient's high‐risk metabolic profile, making timely wound‐bed optimisation particularly important. Importantly, NPWT was applied with a predefined intent as a bridge to reconstruction, aimed at controlling exudate and conditioning the wound bed to enable definitive plastic coverage (STSG), rather than being presented as a universal method to shorten healing time. This distinction is clinically relevant because high‐quality pragmatic evidence in predominantly diabetic lower‐limb SWHSI has not shown a reduction in time to healing with NPWT compared with usual care (SWHSI‐2: *n* = 686; 80.0% diabetic; median 187 vs. 195 days; HR 1.08; *p* = 0.47) [15].

Conversely, when the clinical objective is definitive coverage, NPWT has stronger evidence as an adjunct to STSG: a 2025 meta‐analysis of 16 RCTs (411 NPWT vs. 401 controls) reported higher overall graft take (+8.3%), higher graft success (OR 1.86) and fewer reoperations (OR 0.31) versus conventional dressings, supporting its role in facilitating successful reconstruction in appropriately selected patients [16].

This case also highlights the importance of tailoring postoperative wound care to patient‐specific risk factors.

## 4 Conclusion

NPWT appeared to be a useful adjunctive strategy in a patient with multiple comorbidities. Its application promoted rapid wound healing, minimised infection risk and created favourable conditions for definitive closure with autologous grafting. This case emphasises the value of NPWT in gynaecological surgery and underscores the importance of a multidisciplinary approach in optimising outcomes for high‐risk patients.

## Ethics Statement

In accordance with our institutional policy, formal ethics committee/IRB approval was not required for the publication of this single, fully anonymised case report and patient confidentiality was maintained throughout manuscript preparation.

## Consent

Written informed consent was obtained from the patient for the preparation and publication of this manuscript, including the use and publication of all accompanying clinical images.

## Conflicts of Interest

The authors declare no conflicts of interest.

## Data Availability

Data sharing not applicable to this article as no datasets were generated or analysed during the current study.
